# Assessing HyperSight iterative CBCT for dose calculation in online adaptive radiotherapy for pelvis and breast patients compared to synthetic CT

**DOI:** 10.1002/acm2.70038

**Published:** 2025-03-03

**Authors:** Jingwei Duan, Joel A. Pogue, Dennis N. Stanley, Sui Shen, Natalie N. Viscariello, Carlos E. Cardenas, Richard A. Popple, Joseph Harms

**Affiliations:** ^1^ Department of Radiation Oncology University of Alabama at Birmingham Birmingham USA; ^2^ Department of Radiation Oncology Washington University School of Medicine Saint Louis Missouri US

**Keywords:** dose calculation, HyperSight, image‐guided radiation therapy, online adaptive radiotherapy

## Abstract

**Purpose/objectives:**

Recent technological advancements have increased efficiency for clinical deliverability of online‐adaptive‐radiotherapy (oART). Previous cone‐beam‐computed‐tomography (CBCT) generations lacked the ability to provide reliable Hounsfield‐units (HU), thus requiring oART workflows to rely on synthetic‐CT (sCT) images derived through deformable‐image‐registration (DIR) between the planning CT (pCT) and the daily CBCT. These sCTs are prone to errors stemming from DIR, potentially contributing to dosimetric errors. This study aims to evaluate the capability of direct dose calculation using a novel CBCT platform, HyperSight (Varian‐Medical‐Systems), as an alternative for sCT.

**Methods/materials:**

To validate the HyperSight iterative CBCT (HS‐iCBCT) HU accuracy, 125 kV and 140 kV HS‐iCBCT calibration curves were benchmarked against a pCT calibration curve. To determine the clinical impact of HS‐iCBCT compared to sCT, daily adaptive sessions from 47 oART fractions from 10 patients were analyzed. For these patients, HS‐iCBCT was acquired for daily adaption, and sCT was generated as part of the standard adaptive workflow. After daily adaption, dose was recalculated directly using the HS‐iCBCT, and the HS‐iCBCT and sCT dose distributions were compared by γ‐index and dose‐volume‐histogram (DVH) analysis.

**Results:**

The mean HU differences of pCT minus HS‐iCBCT (140/125 kV) were −40.97/−57.79, 9.86/21.74, and 87.22/158.20 for lung, water, and bone. In the patient cohort, the median gamma passing rates between HS‐iCBCT and sCT‐based dose calculations for 3%/2 mm and 1%/1 mm were 99.57 and 96.45% with 10% threshold, and 99.92% and 86.15% with 80% threshold. Dosimetric deviations in high dose regions were concentrated in areas with larger deformation, that is, surface change and variable bladder/bowel filling. The median (min‐max) D98%/V100% absolute deviations were 0.3(0.0–1.6)/0.0(0.0–13.7) and 0.4(0.0–1.4)/0.5(0.0–17.5) for CTVs and PTVs.

**Conclusions:**

The HS‐iCBCT platform with iterative reconstruction provides dose calculation comparable to sCT for pelvis and breast patients. However, acceptable, yet noticeable, dose discrepancies between HS‐iCBCT and sCT exist, particularly in high‐dose regions. Further investigations are needed to benchmark these methods against ground truth measurements.

## 1 INTRODUCTION

KV cone‐beam computed tomography (CBCT) based online adaptive radiation therapy (oART) has been integrated into the clinic over recent years with the introduction of the Ethos platform (Varian Medical Systems, Palo Alto, CA). During oART, the radiation treatment plan is optimized based on daily patient anatomy, aiming to offer optimal dosimetric outcomes by improving target coverage and normal tissue sparing.^1^ Research has demonstrated improvement in the dosimetry and plan quality across various sites, encompassing the entire body.^2^, ^3^, ^4^, ^5^, ^6^, ^7^, ^8^, ^9^, ^10^, ^11^

In current practice for CBCT‐guided oART using Ethos (v1.1), the treatment plan optimization and dose calculation is performed using a synthetic CT (sCT) image because the daily CBCT does not provide Hounsfield unit (HU) accuracy necessary for dose calculation.^4^, ^7^ The sCT is generated through a series of steps starting with a deformable image registration (DIR) of the planning CT (pCT) to the on‐treatment CBCT. The HUs of the pCT are then propagated according to the deformation vector field (DVF), allowing for a density map reflecting daily anatomy, assuming minimal errors in the DIR. However, sCT images are subject to potential limitations aligned with the nature of DIR; these include truncation and distortion artifacts due to discrepancies in field of view (FOV), limited image length, difference in anatomy between pCT and daily CBCT images, and patient movement.^12^, ^13^ When these artifacts overlap with areas of significant HU changes, such as erroneously substituting air pockets in the bowel with soft tissue, there is potential for dosimetric inaccuracy, and subsequently increased uncertainty in dose delivery. As such, sCT images are limited in their ability to accurately reflect the patient's unique, daily density map, especially in cases of significant organ and patient changes, such as bowel/bladder filling, large weight gain or loss, or large tumor shrinkage/growth. Numerous studies have explored the limitations of sCT on the Ethos platform. Kisling et al. showed the inadequacies of sCT in terms of mapping density changes associated with weight loss and gain.^14^ Additionally, Wegener et al. identified that incorrect density distribution in the sCT has significant impact on dose calculation and optimization in the lungs,^15^ and revealed errors in sCT for bolus‐covered surfaces on anthropomorphic phantom.^16^

Direct optimization and dose calculation on daily kV‐CBCT could mitigate the limitations of the DIR‐generated sCT. In fact, the concept of calculating radiation dose using daily CBCT predates the clinical implementation of sCT.^17^, ^18^ However, the HU inaccuracy, added noise, and limited FOV associated with daily kV‐CBCT have thus far hindered direct dose calculation,^19^ with reported dosimetric discrepancies of up to 6.7% for simple plans.^20^ Some studies have been conducted to correct the HU number of kV‐CBCT by using patient specific,^21^, ^22^ site specific,^23^ and population based^18^, ^19^ methods. However, they are either not robust enough for anatomical regions with large heterogeneity or present challenges for integration into current clinical workflows. Deep learning techniques for sCT generation have also been proposed,^24^, ^25^, ^26^, ^27^ but many of these have yet to be implemented in clinical practice. Additionally, patient motion artifacts (e.g., streaking) in sites such as the abdomen and thorax also hinder direct kV‐CBCT dose calculation,^28^, ^29^ as the patient's breathing cycle is significantly shorter than the typical kV‐CBCT acquisition time (around 40s). Despite various efforts to correct or mitigate motion artifacts in CBCT, including model‐based,^30^ patient‐based (e.g., deep inspiration breath hold), or image reconstruction‐based^28^ approaches, the quality of kV‐CBCT has not achieved the accuracy necessary for direct dose calculation. Finally, the FOV of kV‐CBCT has historically been limited due to the dimensions of the detector panel, which results in incomplete coverage and fails to capture the full body.

The recently‐introduced HyperSight (HS)™ technology (Varian Medical Systems, Palo Alto, CA) provides an alternative solution for oART by seeking to mitigate these limitations, with an extended 70 cm FOV.^31^ This enhancement is facilitated by a larger detector panel and faster gantry rotation speed on a ring‐based linear accelerator system, which significantly reduced, 6‐s CBCT acquisition.^32^ In addition to hardware upgrades, HS provides an iterative reconstruction algorithm enhanced by a grid‐based Boltzmann Solver for x‐ray scatter correction, which has been shown to outperform the standard Feldkamp–Davis–Kress CBCT reconstruction in head and neck cases.^33^ Consequently, this leads to improvement in imaging quality and soft tissue visualization for HyperSight iterative CBCT (HS‐iCBCT).^34^ A recent study by Robar et al illustrated that HS provides less severe artifacts compared to its predecessors, with statistically similar artifact index values compared to simulation CTs.^32^, ^35^ Preliminary investigations and manufacturer technical reports both stated the superior imaging quality over previous platforms.^32^ Therefore, these technical improvements potentially facilitate direct dose calculation on HS‐iCBCT images.

Given the reported improvements HyperSight can offer, it is crucial to evaluate the dosimetric feasibility of direct HS‐iCBCT dose calculation in the Ethos oART workflow before clinical implementation. While a recent study has explored the accuracy of HS in comparison to pCT using anthropomorphic phantoms, dosimetric evaluation in real patient cases, one of the prerequisites for clinical implementation remains unexplored.^36^ Another group evaluated HS imaging performance and showed an increase in image quality and noise reduction of HS imager compared to previous imagers.^37^ However, despite its superior imaging performance compared to its predecessor, the capability for direct dose calculation on HS‐iCBCT remains unclear. Here we present a comprehensive evaluation of the dosimetric performance of direct HS‐iCBCT dose calculation in pelvis and breast oART patient treatments, comparing these to sCT, the current standard of care.

## 2 METHOD AND MATERIALS

### 2.1 HU accuracy verification and calibration curve establishment

Although the image quality of HS‐iCBCT has been assessed previously,^32^ it is essential to start the validation process with in‐house verification of HU accuracy, which is one of the fundamental assumptions of direct dose calculation on HS‐iCBCT images. In this study, we assessed the HU accuracy of HS‐iCBCT against pCT by using a Gammex 467 tissue characterization phantom with fourteen tissue substitute materials. The physical densities of tissue substitute materials ranged from 0.01 (Air) to 4.59 (Titanium) g/cm^3^. HS‐iCBCT images were acquired using two scanning protocols: iCBCT (125 kV) and iCBCT (140 kV). Similarly, the tissue characterization phantom was scanned using a Phillips Big Bore CT simulator for paired comparison, following institutional pelvis imaging protocol (120 kVp, 20 mAs, and 3 mm slice reconstruction). Mean and standard deviation values for each tissue insert were extracted using from a cylindrical region of interest (ROI) with a radius of 5 mm and height of 18 mm, centered on each insert. Subsequently, the calibration curves from both iCBCT protocols were compared against the established the pCT calibration curve from treatment planning system (TPS).

### 2.2 Dosimetric evaluation

This retrospective dose calculation study was covered under an Institutional Review Board approved protocol (IRB‐120703005). Following a clinical upgrade to the HyperSight image guidance system on our Ethos treatment delivery platform, datasets from 47 oART fractions for 10 patients were acquired with either 140 kV (*n* = 12) or 125 kV (*n* = 35) scanning protocols. HS‐iCBCT images were acquired during oART treatment and subsequently utilized to generate sCT images. These patients received oART treatments which were optimized using sCT in the standard workflow.^7^ In the multi‐step image registration process required for sCT generation, the pCT images were first rigidly registered to HS‐iCBCT images before DIR. Therefore, HS‐iCBCT and sCT images share the same coordinate system and DICOM frame of reference. Daily contours were then generated directly on the HS‐iCBCT and directly transferred to the sCT. After treatment, all session data, including HS‐iCBCT and sCT images, daily contours, and treatment plans, were transferred from the Ethos TPS to Eclipse (Varian Medical Systems, Palo Alto, CA) TPS for dose recalculation and comparison. Five fractions, evenly distributed throughout the patient course following the clinical upgrade to Hypersight were selected for each patient, except for one male pelvis patient who only had two fractions remaining after the implementation. The patient cohort included six pelvis patients (two female and four male pelvis patients), and four accelerated partial breast irradiation (APBI) breast patients (two right and two left). These were the first 10 patients treated within the first month following clinical implementation of HyperSight, without other specific selection criteria.

Dosimetric comparison between HS‐iCBCT and sCT images was performed by directly recalculating the dose on HS‐iCBCT and subsequently comparing the dose distribution to the sCT for each fraction. To simulate scenarios of dose calculation using HS‐iCBCT only, the body contours for HS‐iCBCT images were regenerated and visually verified for each image. It is noted that Ethos v2.0 only allows for one calibration curve to be used for HS‐iCBCT, and our dataset contains more 125 kV scans than 140 kV scans. To simulate the future application of HS‐iCBCT for dose calculation with its specific calibration curve, HS‐iCBCT dose calculations were conducted using the HS‐iCBCT (125 kV) HU calibration curve. In contrast, the sCT dose calculations utilized the pCT HU calibration curve, which serves as the current standard. Additionally, all HS‐iCBCT and sCT dose calculations were performed with same plan settings in Eclipse using Acuros XB (AXB, version 16.10), a grid‐based Boltzmann equation solver.

Dose distributions were exported, and three‐dimensional gamma (γ) index calculations were performed in Matlab using an open‐source 3D gamma calculation script.^38^ Gamma analysis was performed with using the following combination of criteria: 1%/1 mm, 2%/2 mm, and 3%/2 mm for global dose difference/distance to agreement (DTA), with dose thresholds of 10% and 80%. Calculations with 3%/2 mm and 10% dose threshold were selected as these are the standard for patient‐specific QA in our clinic,^39^ and the stricter criteria analyses were selected to highlight areas of discrepancy between the sCT and HS‐iCBCT‐calculated dose distributions. The γ calculations were performed with a limited search region for DTA of 3 mm. The mean passing rate, minimum passing rate, and mean gamma index with different criteria were recorded. Dose volume histogram (DVH) values of both targets and organs at risk (OARs) were extracted from the TPS. The dose to 98% (D98%) and volumes receiving 100% of prescription dose (V100%) for both the clinical target volumes (CTV) and the planning target volumes (PTV) were analyzed in all cases. In the pelvis, the D0.03cc (Gy) and V45Gy (%) for rectum and bladder were compared. For APBI cases, the ipsilateral breast V15Gy (%), ipsilateral lung V9Gy (%), heart V1.5Gy (%), and skin D0.01cc (Gy) were included. Given the variance in prescriptions across disease sites, all doses are reported relative to the prescription dose.

## 3 RESULTS

**FIGURE 1:**
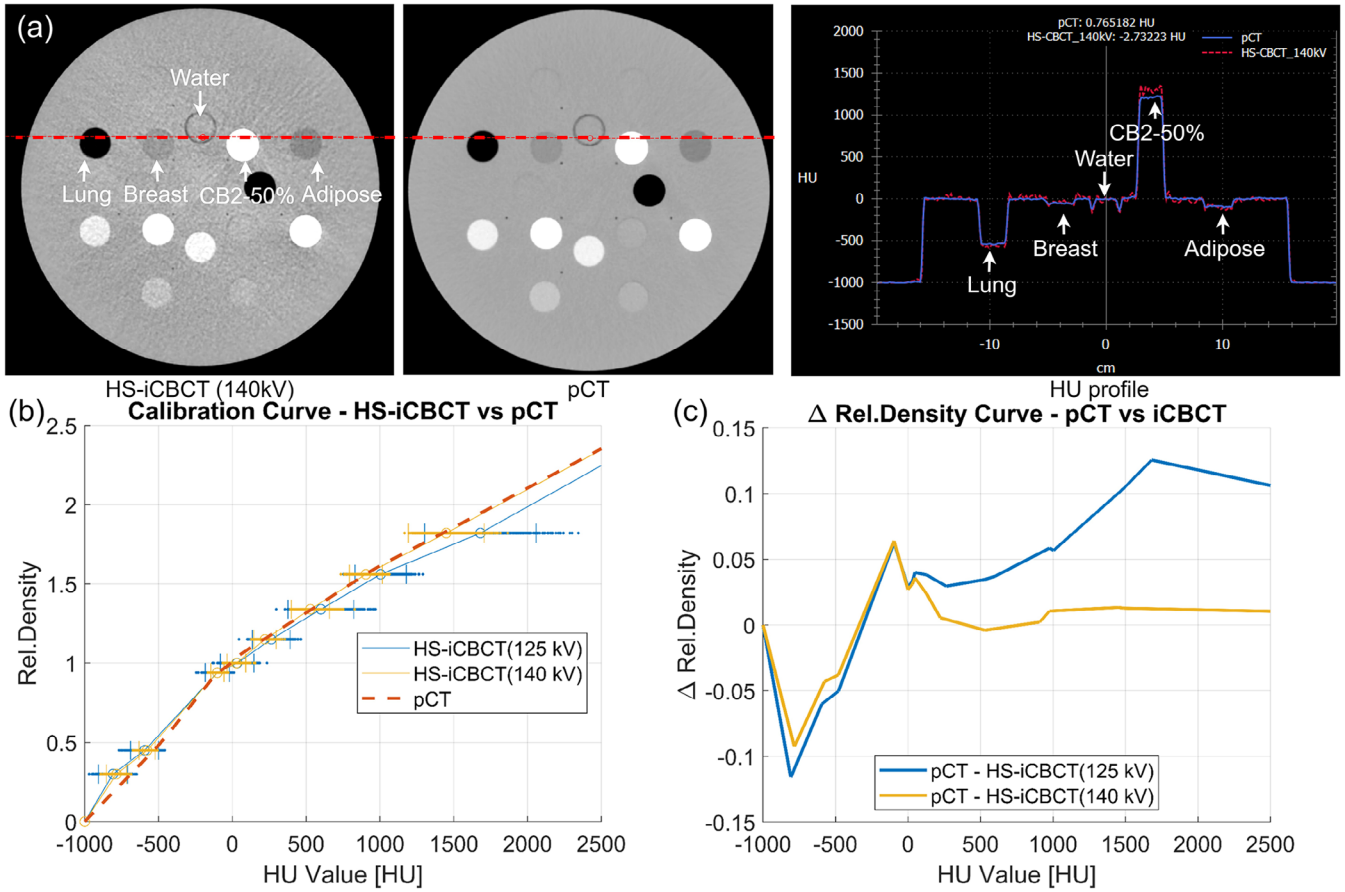
(a) The example slices of HS‐iCBCT and pCT images for Gammex 467 tissue characterization phantom. The dotted red line on the CT images is represented by the line profile on the right. Images shown on the HU window [−160, 240]. (b) The calibration curves for both HS‐iCBCT (125 and 140 kV) and pCT. (c) The differences between calibration curves. HU, Hounsfield‐units; HS‐iCBCT, HyperSight iterative CBCT; pCT, planning CT.

Table 1 summarizes the HU measurement for various tissue/implant substitute materials, comparing pCT with HS‐iCBCT at 140 kV and 125 kV. The deviations among common human body tissues, ranging from lung to bone (B‐200), are within 100 HU at 140 kV and 200 HU at 125 kV. The largest deviations are −269.7 HU for Titanium at 140 kV and 473.51 HU for CB2‐50% at 125 kV. Additionally, the standard deviations at 140 kV are consistently lower than those at 125 kV for the same materials, indicating less noise at the higher voltage. Note that the pCT data is 16‐bits while HS is 13‐bits, meaning that the maximum theoretical value reportable by the pCT is 64 536 HU, while for the HS‐iCBCT it is 7 192 HU. Practically, we found that the titanium insert of the phantom had a maximum HU of 7 000. This contributes to the larger HU deviation for Titanium, with an HU near the maximum range. Figure 1(a) shows the HU profile across some tissue substitute materials, with a physical density range from 0.45 (LN‐450) to 1.82 (CB2‐50%) with HS‐iCBCT at 140 kV. The calibration curves were established from the HU number measured from tissue characterization phantom with fourteen tissue substitute materials. Figure 1(b) indicates the calibrations curve of both HS‐iCBCT at 140 and 125 kV. Figure 1(c) illustrates the density differences between pCT and HS‐iCBCT. The greatest differences are within 0.10 g/cm^3^ for 140 kV HS‐iCBCT, and 0.12 g/cm^3^ for 125 kV HS‐iCBCT. The HU accuracy of titanium was also evaluated, but is outside the illustrated data domain.

**TABLE 1:** The HU measurement on the Gammex 467 tissue characterization phantom with 14 tissue substitute materials by pCT and HS‐iCBCT at 140 kV and 125 kV.

		pCT	HS‐iCBCT(140 kV)		HS‐iCBCT(125 kV)	
	Physical Density (g/cm^3^)	Measured HU	Measured HU	Deviation	Measured HU	Deviation
Lung (Ln‐450)	0.45	−536.7 ± 14.7	−577.7 ± 27.8	−41.0 ± 31.5	−594.5 ± 48.4	−57.8 ± 50.6
Adipose	0.94	−91.8 ± 5.7	−101.0 ± 22.6	−9.2 ± 23.3	−101.8 ± 41.7	−10.0 ± 42.1
Breast	0.98	−49.6 ± 6.7	−42.3 ± 28.0	7.3 ± 28.8	−33.7 ± 54.3	15.9 ± 54.7
Water	1	0.0 ± 7.1	9.9 ± 32.1	9.9 ± 32.8	21.9 ± 58.0	21.9 ± 58.4
Solid water	1.02	2.1 ± 6.3	27.9 ± 30.8	25.9 ± 31.4	32.5 ± 54.4	30.5 ± 54.7
Muscle	1.05	24.4 ± 10.5	49.3 ± 32.6	24.9 ± 34.3	61.6 ± 62.8	37.2 ± 63.6
Brain	1.1	86.4 ± 6.3	79.8 ± 31.3	−6.6 ± 31.9	93.3 ± 60.2	6.9 ± 60.5
Liver	1.14	217.8 ± 7.3	269.8 ± 42.3	52.1 ± 43.0	301.6 ± 79.6	83.8 ± 79.9
Inner Bone	1.15	213.5 ± 6.9	220.8 ± 38.73	7.3 ± 39.3	263.9 ± 65.33	50.4 ± 65.7
Bone (B‐200)	1.34	441.4 ± 8.2	528.6 ± 65.7	87.2 ± 66.2	599.6 ± 113.7	158.2 ± 113.9
CB2‐30%	1.56	810.0 ± 7.3	904.7 ± 56.7	95.1 ± 57.2	1004.5 ± 88.7	194.9 ± 89.0
CB2‐50%	1.82	1206.8 ± 8.9	1449.3 ± 130.9	242.5 ± 131.2	1680.3 ± 192.7	473.5 ± 192.9
Titanium	4.59	7255.6 ± 270.7	6985.9 ± 73.2	−269.7 ± 280.4	7000 ± 0.0	−255.6 ± 270.7

Table 2 summarizes the 3D gamma index calculation results with different criteria between HS‐iCBCT and sCT‐based dose distribution for 47 images. Using the strictest criteria (1%/1 mm, 80% threshold), large dosimetric discrepancies were observed in the high dose regions. Across all plans (*n* = 47) with the strictest criteria, the mean gamma passing rate was reported as 86.31 ± 6.24% with mean gamma indices of 0.52 ± 0.11%. For site specific analysis, pelvis plans (*n* = 27) reported a mean passing rate of 88.04% ± 6.01% and mean gamma indices of 0.49 ± 0.11, and breast plans (*n* = 20) yielded mean passing rates of 83.97% ± 5.91% with mean gamma indices of 0.57 ± 0.11. When applying a less strict criteria of 3%/2 mm and 80% threshold, the gamma passing rate increased to 99.90 ± 0.18% for pelvis plans and 98.85 ± 1.92% for breast plans. Among all plans evaluated using 3%/2 mm and an 80% threshold, only two breast plans had gamma passing rates below 95%, specifically 94.02% and 94.47%.

**TABLE 2:** Summary of gamma calculation results between the HS‐iCBCT and sCT‐based dose distributions. Data presented are the mean ± standard deviation values over 47 evaluated cases.

			10% threshold			80% threshold	
		3%/2 mm	2%/2 mm	1%/1 mm	3%/2 mm	2%/2 mm	1%/1 mm
Total (*n* = 47)	Mean passing rate	99.57 ± 0.30%	99.36 ± 0.41%	96.06 ± 1.75%	99.45 ± 1.35%	98.47 ± 1.91%	86.31 ± 6.24%
	Min passing rate	98.89%	98.45%	92.16%	94.02%	90.66%	67.03%
	Mean gamma index	0.13 ± 0.02	0.16 ± 0.02	0.29 ± 0.05	0.21 ± 0.05	0.27 ± 0.06	0.52 ± 0.11
Pelvis (*n* = 27)	Mean passing rate	99.46 ± 0.26%	99.20 ± 0.37%	95.40 ± 1.60%	99.90 ± 0.18%	99.06 ± 0.87%	88.04 ± 6.01%
	Min passing rate	98.89%	98.51%	92.16%	99.28%	96.61%	78.18%
	Mean gamma index	0.14 ± 0.02	0.16 ± 0.02	0.31 ± 0.04	0.19 ± 0.03	0.25 ± 0.05	0.49 ± 0.11
Breast (*n* = 20)	Mean passing rate	99.73 ± 0.28%	99.57 ± 0.37%	96.94 ± 1.57%	98.85 ± 1.92%	97.66 ± 2.56%	83.97 ± 5.91%
	Min passing rate	98.97%	98.45%	92.59%	94.02%	90.66%	67.03%
	Mean gamma index	0.12 ± 0.02	0.14 ± 0.02	0.27 ± 0.05	0.23 ± 0.05	0.30 ± 0.06	0.57 ± 0.11

**FIGURE 2:**
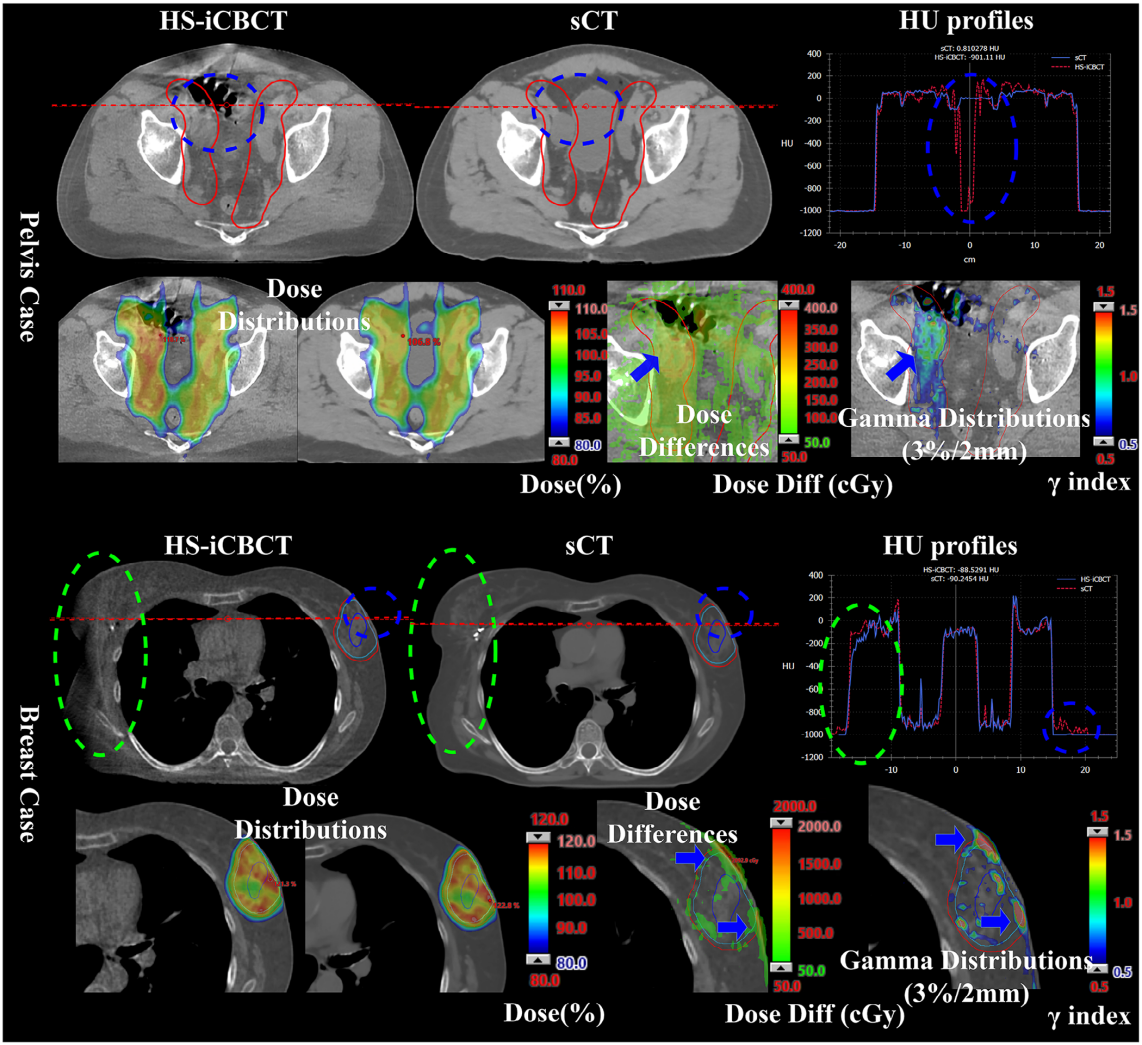
The example slices, HU profiles, dose distributions, dose differences, and gamma distributions for two example cases from pelvis and breast sites. Blue arrows highlight the distortions of sCT and following dosimetric errors. Blue dash circle highlights the failure of sCT to monitor the patient daily anatomical variation and following dosimetric errors. Green dash circles highlight the limited FOV of HS‐iCBCT, noticeable in areas far from the isocenter. FOV, field of view; HU, Hounsfield‐units; HS‐iCBCT, HyperSight iterative CBCT; sCT, synthetic‐CT.

Figure 2 shows example CT images, HU profiles, dose distributions, dose differences, and gamma distributions from two adaptive fractions, one for each site. Blue dashed circles highlight material discrepancies between the HS‐iCBCT and sCT images. The sCT struggled with several issues: adapting to bowel and bladder filling in the pelvis case, and detecting surface changes in the breast case, which were successfully captured by HS‐iCBCT. However, the HS‐iCBCT was challenged by a limited FOV, as indicated by the green dash circle in breast case. This limited FOV is due to the isocenter typically being placed at the center of the PTV, which can be offset from midline by several cm for breast patients. For the individual pelvis and breast fractions shown in Figure 2, the gamma passing rates (using an 80% threshold) were 78.18%, and 67.03% for 1%/1 mm as well as 99.68%, and 94.02% for 3%/2 mm criteria, respectively. In pelvis cases, the sCTs did not reflect variations in bladder filling seen in the HS‐iCBCTs, producing local dose calculation discrepancies of up to 4.8%. For APBI patients, the target area, located up to 3 mm proximal to the body surface, was influenced by surface variations relative to simulation, leading to dose differences in surface regions. Additionally, the HS‐iCBCT image was unable to fully capture the density information for areas distant from the isocenter, as indicated by the green dash circle.

**FIGURE 3:**
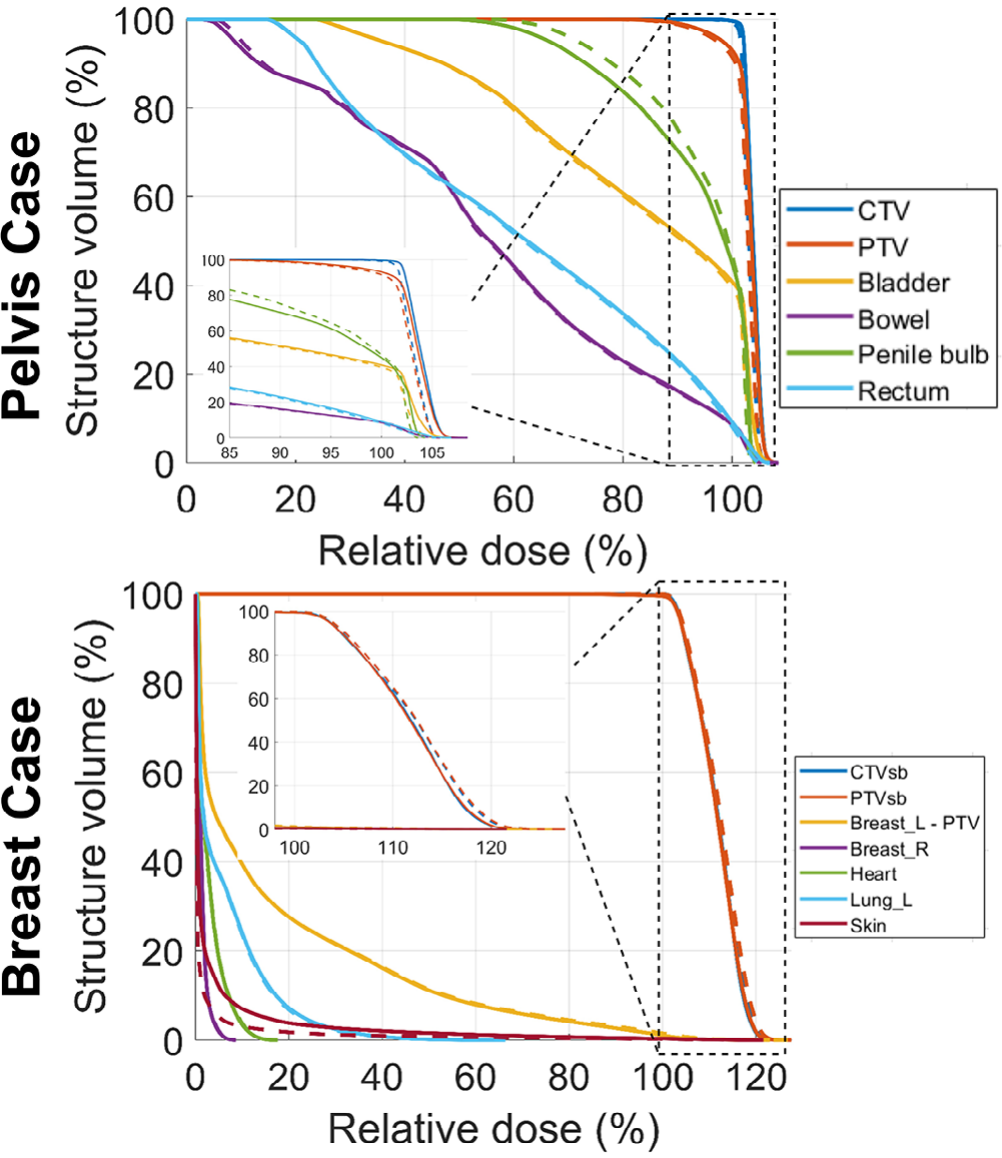
The DVH for two example cases from pelvis and breast sites. The solid lines mean DVH profiles from HS‐iCBCT plan, while the dash lines indicate DVH profiles from sCT plan. DVH, dose volume histogram; HS‐iCBCT, HyperSight iterative CBCT; sCT, synthetic‐CT.

**FIGURE 4:**
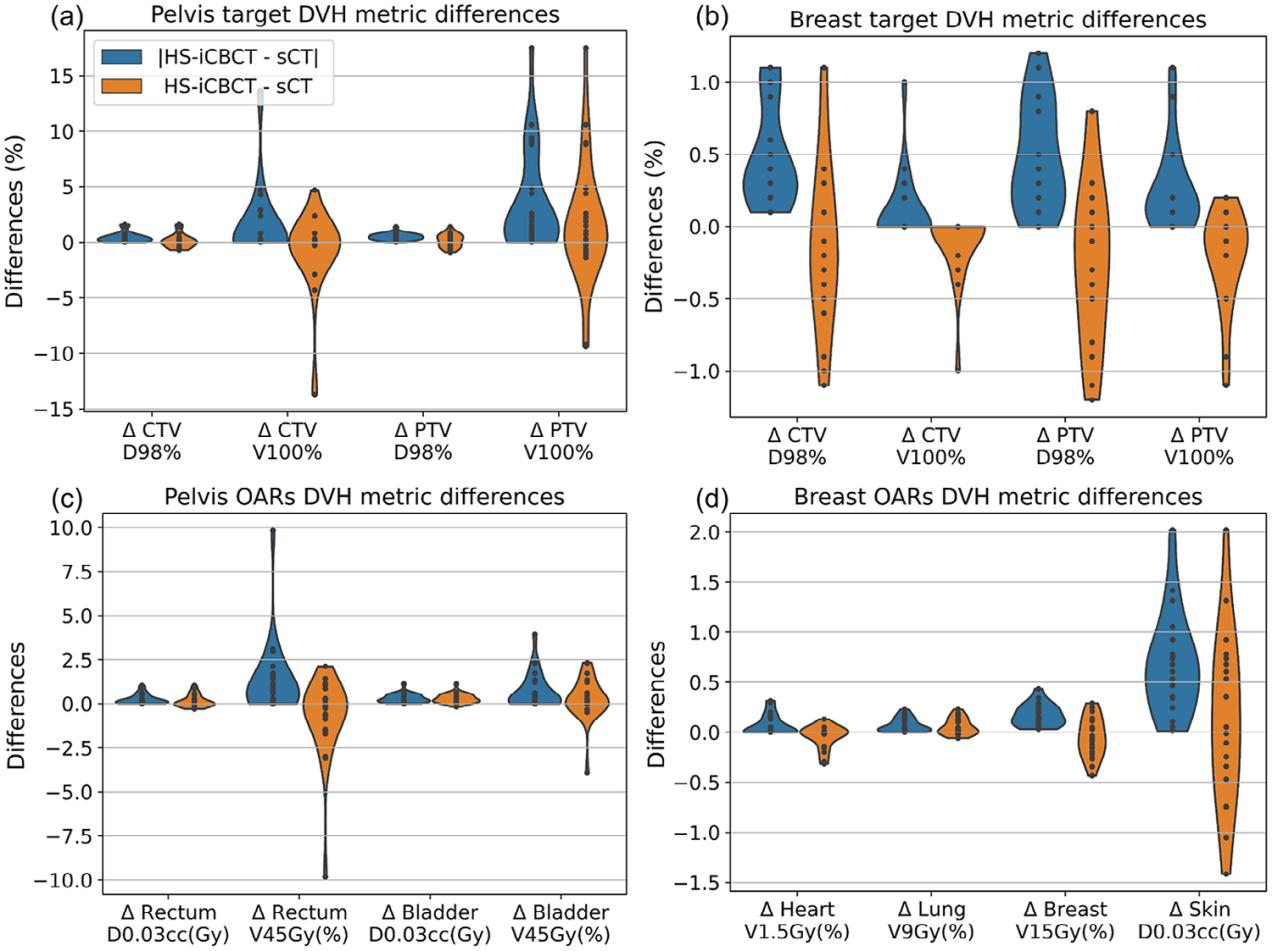
The absolute and relative targets (a), OARs in pelvis cases (b), and OARs in APBI breast (c) cases DVH metric differences in dose calculation between HS‐iCBCT and sCT. APBI, accelerated partial breast irradiation; DVH, dose volume histogram; HS‐iCBCT, HyperSight iterative CBCT; oART, online‐adaptive‐radiotherapy; sCT, synthetic‐CT.

Figure 3 shows the DVH for example cases illustrated in Figure 2. The DVHs exhibit differences between HS‐iCBCT and sCT for one or more targets or OARs. Noticeable DVH differences were observed in the targets (CTV and PTV) and penile bulb for the pelvis case, and the skin for the breast case. The absolute and relative (HS‐iCBCT minus sCT) DVH differences for both CTVs and PTVs are shown in Figure 4(a–b). The largest discrepancy was observed in the PTV V100, with differences in mean and median values of 2.06% and 0.50%, respectively. The maximum discrepancy observed for PTV V100 was 17.5%. Figure 4(c‐d) illustrates the absolute and relative DVH differences (HS‐iCBCT minus sCT) for pelvis and APBI breast cases. The largest observed discrepancies were 9.8% for rectum V45Gy in pelvis cases and 2.0% for skin D0.03cc in APBI breast cases. Overall, DVH analysis for both targets and OARs revealed noticeable differences in dose calculations between HS‐iCBCT and sCT.

## 4 DISCUSSION

With the integration of HyperSight into the clinical workflow, there is interest in the CBCT‐guided oART community toward direct reliance on HS‐iCBCT for optimization and dose calculation, transitioning away from the current standard of care, sCT. This study assesses the feasibility of direct dose calculation using HS‐iCBCT and compares dose calculations to sCT on a retrospective oART dataset. Our findings align with Robar et al.’s assessment of image quality on the HS platform, showing a similar performance of pCT in terms of HU accuracy.^32^ Even though daily CT simulations, which can serve as ground‐truth density maps, are not available in this study, the HS platform has demonstrated comparable dosimetric accuracy to pCT in anthropomorphic phantoms by Bogowicz et al.^36^ In this study, gamma analysis was first performed with criteria of 3%/2 mm and a 10% dose threshold, in line with the recommendations for patient‐specific QA outlined by AAPM TG‐218.^39^ Considering a plan to be within tolerance limits if >95% of voxels had a gamma value less than or equal to 1, we found that all plans passed the gamma analysis, suggesting that direct dose calculation using HS‐iCBCT is comparable to using sCT for both pelvis and breast patients.

Further tests were conducted with stricter criteria at both 2%/2 mm and 1%/1 mm, as well as increasing the dose threshold to 80% of prescription dose. This analysis was conducted to highlight areas of disagreement, and these results are shown in Table 2. We found that disagreements between sCT and HS‐iCBCT, and corresponding dosimetric discrepancies, were localized to areas with (1) significant anatomical variations, (2) targets close to regions with high anatomical heterogeneity, and (3) body surfaces. The noted difference is a combination of all uncertainties and limitations from both sCT and HS‐iCBCT.

In Figure 4(a–b), noticeable differences in target DVHs were observed between HS‐iCBCT and sCT, particularly in pelvis cases. Through visual inspection on cases with large differences, this discrepancy may be explained by (1) the presence of differing air pockets between the HS‐iCBCT and sCT in pelvis cases, and (2) the plans were optimized on sCT instead of HS‐iCBCT. For pelvis cases, the sCT displayed air pockets like those seen in the pCT, while the daily anatomy from HS‐iCBCT revealed varying air pockets. Additionally, the V100% target DVH metric is particularly sensitive to these significant HU differences (air vs. normal tissue). For APBI cases, the systematically lower CTV V100% can be attributed to the fact that plans were optimized to achieve nearly 100% for CTV V100% on sCT. However, the different density map from HS‐iCBCT made it challenging for the CTV to be fully covered by the 100% isodose line when using a plan optimized on sCT.

Problems inherent to DIR‐generated sCTs have been shown previously, leading to subsequent dosimetric inaccuracies.^14^, ^15^, ^40^ Wegner et al. demonstrated that the growth or shrinkage of simulated tumor cannot be correctly reflected by sCT in an anthropomorphic lung phantom study.^15^ They reported that PTV median dose differences of up to 14.7% when replacing a 3 cm diameter insert in the pCT with a 1 cm insert at the time of treatment. Additionally, in the pelvis case shown in Figure 2, we observed a similar phenomenon where sCT generated an inaccurate density map because of bladder and bowel variation. The magnitude of presented dosimetric inaccuracies would likely be amplified in conditions of large heterogeneity. Recently, Duan et al. reported a gamma passing rate of 72.75% when comparing sCT with same‐day pCT using the same plan parameters in a lung case study with significant tumor regression.^40^ These limitations of the sCT are problematic because, in theory, patients with these large anatomical changes have the highest potential clinical benefits from oART.

HS‐iCBCT also has its limitations when implemented for oART. First of all, its FOV remains limited, especially when the treatment isocenter is lateral on the patient, which could result in missing density information and potentially affect dose calculation. However, dose errors due to missing density information in the breast example case from Figure 2 were minimized because it was treated with partial arcs which did not enter near the incomplete CT data. Yet, it should be emphasized that the resulting dose uncertainties will increase if treatment fields enter areas of missing density information. Additionally, it is challenging to determine whether sCT or HS‐iCBCT provides a more accurate external body contour when the FOV is limited and significant artifacts are present. Therefore, users should pay additional attention to cases involving full arc VMAT or intensity‐modulated radiation therapy when treating far from midline.

CBCT streaking artifacts, illustrated in Figure 2, persist and remain a concern for HS‐iCBCT image quality. Robar et al also revealed similar issues remain, even though performance is improved over conventional gantry‐mounted CBCT.^32^ Traditionally, HS‐iCBCT tends to be noisier than pCT. In this study, we utilized separate calibration curves for sCT and HS‐iCBCT, although users could implement a pCT calibration curve for HS‐iCBCT dose calculation if desired. Our study reported that HS‐iCBCT HU standard deviations were lower than 100 HU for most tissues equivalent materials, aligning with findings from a parallel study.^37^ In a study correlating dosimetry changes with HU changes, Davis et al. revealed that the HU change in soft tissue has the greatest impact on dose variation.^41^ Specifically, they observed dose variations in PTVs and OARs below 0.5% and 1% when HU changes were within ± 20 HU for soft tissue and ±50 HU for bone and air, respectively.

The imaging protocols employed in this study were HS‐iCBCT at 140 kV and 125 kV. The vendor also provides additional imaging protocols, such as HS‐iCBCT at 100 kV (head‐and‐neck imaging protocol), and HS‐FDK, which currently do not support direct dose calculation and were therefore excluded from our analysis. As illustrated in Figure 1 and Table 1, the HU deviations are more pronounced at 125 kV, particularly for materials with high density. The imaging threshold tool tends to segment a broader area of high‐density material than what is displayed on pCT around high‐density objects, suggesting the potential over‐contouring of implanted materials, such as titanium, in HS‐iCBCT images. The Ethos v2.0 platform will automatically outline high‐density regions (density equal or above 2800 HU) and assign titanium as the material if no density override is performed manually. Research has reported the dosimetric uncertainty associated with improper density override.^42^ Additionally, the HS platform's 13‐bit imaging, with an HU range of −1000 to 7192, does not differentiate between titanium and materials of higher density (such as stainless steel), suggesting the necessity to propagate density overrides from pCT to HS‐iCBCT to ensure precise dose calculation.

This study primarily investigates the dosimetric differences between HS‐iCBCT and sCT when using identical plans. In the current oART workflow, daily adaptive plans are optimized on sCT images. The relationship between dose calculation and optimization is dynamic and interdependent.^43^ Thus, the impact of changing from sCT to HS‐iCBCT for optimization on final plan quality and accuracy remains to be determined.

Recent research has shown promising results for HS‐iCBCT in cases with more tissue heterogeneity and tumor regression. For instance, a recent lung case study comparing dose calculations on HS‐iCBCT and sCT demonstrated that HS‐iCBCT aligned more closely with same‐day pCT, which served as the ground‐truth.^40^ The finding suggests that HS‐iCBCT may offer advantages in such scenarios. However, a recent study also found the potential challenges for HS‐iCBCT dose calculation in lung cases related to breathing motion.^44^ Without ground‐truth images in this study, it is challenging to determine which dose calculation, HS‐iCBCT or sCT, would result in fewer dosimetric errors. Nevertheless, these results indicate that the quality of direct dose calculation on HS‐iCBCT is at least comparable to sCT following the scope of TG‐218, suggesting HS‐iCBCT is capable of directly calculating dose for pelvis and breast oART patients. As mentioned, the scope of this study was limited, such as the number of treatment sites and patients involved. Therefore, future dosimetric investigations should be conducted to assess the broader implications of direct dose calculation using HS‐iCBCT.

Meanwhile, the HS platform may benefit other aspects of oART. Currently, patients undergoing oART experience considerable delays on the couch for pre‐treatment preparation, such as contouring edits and plan review.^31^ The auto‐segmentation tool has been utilized in CBCT to mitigate the demand of clinical resources and time. Often, auto‐segmented contours still need edits before re‐optimization and re‐calculation can begin. Moazzezi et al. has reported that 96% of fractions required modification on auto‐segmentation, although they were minor,^45^ and the average contour editing time has been reported at 20 min.^46^ The higher image quality of HS‐iCBCT may enhance the performance of auto‐segmentation, potentially improving the efficacy of oART. Furthermore, the exploration of dose prediction on CT images may also be transferred to HS‐iCBCT images, potentially streamlining the decision‐making process during plan review.

## 5 CONCLUSION

The HyperSight CBCT imaging platform, with an iterative reconstruction algorithm corrected for x‐ray scatter, enables similar dosimetric distributions compared to those generated via sCT, the current standard of care, for breast and pelvis patients. However, acceptable, yet noticeable, dosimetric discrepancies exist due to uncertainties from both sCT and HS‐iCBCT. While sCT provides adequate imaging information for optimization and calculation for most cases, it does not fully capture daily anatomy changes as seen on HS‐iCBCT, such as variable organ filling or changes in body habitus, limiting the utility of oART in a cohort of cases where it could theoretically provide the most benefit. The uncertainties of limited FOV and streaking artifacts from HS‐iCBCT still remain a concern, making further investigations necessary to fully explore these differences and their potential clinical impacts. Moreover, HS‐iCBCT was found to better represent patient anatomy than sCT, particularly when there were large changes between pCT and daily CBCT images.

## AUTHOR CONTRIBUTIONS

The authors contributed to the paper as follows: study design: Jingwei Duan, Joel A. Pogue, Dennis N. Stanley, Joseph Harms; data analysis: Jingwei Duan, Joel A. Pogue, Joseph Harms; measurement data collection: Jingwei Duan, Sui Shen, Joseph Harms; manuscript preparation and revision: Jingwei Duan, Joel A. Pogue, Dennis N. Stanley, Sui Shen, Natalie N. Viscariello, Carlos E. Cardenas, Richard A. Popple, Joseph Harms. All authors reviewed the results and approved the final version of the manuscript.

## CONFLICT OF INTEREST STATEMENT

Dennis N. Stanley has received research support (NIH‐L30 CA284339), and speaker honoraria from Varian medical systems. Richard A. Popple has received research support, not related to this work, and speaker honoraria from Varian medical systems.

## DECLARATION

During the preparation of this work the authors used ChatGPT to assist in proofreading. After using this tool, the authors reviewed and edited the content as needed and takes full responsibility for the content of the publication.
